# Hyberbaric oxygen increases atresia in normal & steroid induced PCO rat ovaries

**DOI:** 10.1186/1477-7827-10-11

**Published:** 2012-02-06

**Authors:** Alev Atis, Yavuz Aydin, Filiz Ciftci, Damlanur Sakız, Abdullah Arslan, Akın S Toklu, Melahat Donmez, Nimet Goker

**Affiliations:** 1 Sisli Etfal Training & Research Hospital Obstetrics & Gynecology, Istanbul, Turkey; 2 Istanbul University Medicosocial Unit Department of Obstetrics & Gynecology, Istanbul, Turkey; 3 Sisli Etfal Training & Research Hospital Department of Pathology, Istanbul, Turkey; 4 Istanbul University Faculty of Medicine, Department of Underwater and Hyperbaric Medicine, Istanbul, Turkey

**Keywords:** Hyperbaric-oxygen, Polycystic-ovary, Rat, Estradiol valerate

## Abstract

**Background:**

In this study, we investigated the effect of hyperbaric oxygen therapy (HBOT) on the morphology of estradiol valerate (EV) induced polycystic ovary (PCO) to find a new treatment modality for improvement of PCO.

**Methods:**

The rats were divided into four groups. Group1, control; group 2, PCO group; group 3, PCO with HBOT group and group 4, normal ovary with HBOT. PCO was induced by a single intramuscular injection of 4 mg EV in adult cycling rats. Other rats with normal ovaries had oil injection as placebo. HBOT was applied to third and fourth groups for six weeks. Histopathologic evaluation of ovaries of all groups were performed & compared.

**Results:**

Six weeks of HBOT was resulted in increase in follicular atresia, decrease in the number of primary, secondary, tertiary follicles and decrease in the number of fresh corpus luteum in normal rat ovary. HBOT on polycystic rat ovary, resulted in significant increase in atretic follicles which were already present.

**Conclusions:**

HBOT of six weeks itself, changed ovarian morphology in favor of atresia both in PCO group and control group. This result of aggravated follicular atresia after HBOT on EV induced PCO may be due to long-term exposure in our protocol which with this state seems to be inapplicable in the improvement of PCO morphology.

## Background

Polycystic ovary syndrome (PCOS), the most common endocrinopathy in women of reproductive age is a multifactorial metabolic disease associated with insulin resistance [1]. PCOS is a common condition with a range of clinical features. These reproductive features include anovulation, irregular menstrual cycles, clinical and biochemical hyperandrogenism and infertility. Metabolic features include increased risk factors for type 2 diabetes mellitus and cardiovascular disease and increase in the prevalence of the metabolic syndrome [1,2].

Previously, the diagnosis of PCOS was based on the National Institute of Health (NIH) criteria comprising biochemical or clinical hyperandrogenism and anovulatory irregular menstrual cycles with the exclusion of related reproductive disorders. In 2003, diagnostic guidelines for PCOS were expanded to the so-called Rotterdam criteria, now based on presentation with any two of the three criteria of hyperandrogenism, irregular anovulatory periods or polycystic ovaries on ultrasound [1,3]. The reported prevalence range of PCOS is between 2.2% to 26% [1,3,4]. The wide range of estimated PCOS prevalence can be explained by different recruitment processes of the study populations, selection biases, ethnic and racial variations in addition to, the criteria used for its definition and the screening methods used to identify each criteria; considering the Rotterdam versus NIH criteria increase the PCOS prevalence by 2 times. The latest study by Tehrani et al. demonstrated that the prevalence's of PCOS using the NIH definition were 8.5% [4].

The abnormalities detected in PCOS have been attributed to primary defects in the hypothalamic-pituitary-adrenal axis, the ovarian microenvironment, the adrenal gland and the insulin/insulin-like growth factor metabolic regulatory system [2,5]. There is evidence that both hypothalamic and pituitary mechanisms contribute to the gonadotropin dysfunction in PCOS.

The reproductive systems of human beings and other vertebrates are grossly similar [6]. Although the rat is a polytocous rodent, the female has a regular ovarian cyclicity of 4 or 5 days, with distinct proestrus, estrus, and diestrus phases. PCO can be experimentally produced in the rat, that species is a good model for studying the pathophysiology of human PCO by a single dose of the long-acting estrogen, estradiol valerate (EV) which leads to anovulatory state in 13-15 week-old rats [6,7].

The therapeutic application of hyperbaric oxygen (HBO) has been known over three decades in different fields of medicine. Hyperbaric oxygen therapy (HBOT) is defined by the Undersea and Hyperbaric Medical Society (UHMS) as a treatment in which a patient intermittently breathes 100% oxygen while the treatment chamber is pressurized to a pressure greater than sea level (1 atmosphere absolute, ATA). The therapeutic principle behind HBO stems from increasing the partial pressure of oxygen in the tissues of the body. The effects of HBO are based on the gas laws, and the physiological, biochemical effects of hyperoxia [8,9]. High tissue levels of oxygen stimulate angiogenesis by increasing the production of cytokines and many growth factors, especially VEGF. Because of these effects on angiogenesis and hyperoxia, it is a unique method used in the treatment of various illnesses and clinical conditions, such as carbon monoxide poisoning, decompression sickness, osteomyelitis, even diabetic foot and ischaemic wounds [9,10].

Besides, wound healing, HBOT has been used for many clinical treatments. Previous experiments have demonstrated that HBO improves survival in anesthetized rats during heatstroke by reducing multiorgan dysfunction [11]. Furthermore, microarray analysis of gene expression in rat cortical neurons exposed to HBO showed that 17 genes changed in response to all exposure conditions [12]. HBOT is also used for primary liver nonfunction as it stimulated hepatocytes to proliferate. Experimentally, HBO was found significantly effective in the survival of transplanted cells or tissues like liver, bone, thyroid, pancreatic islet cells [13]. Although a large proportion of ovarian follicles were lost during the initial ischemia after transplantation of ovaries, recently it was also found that HBO was also beneficial for the follicular survival of the transplanted mouse ovaries [14].

HBOT was also used in invitro-fertilisation (IVF) protocols in different studies for improving ovarian folicular stimulation and endometrial receptivity as an adjunct to infertility therapies with the hypothesis of increasing impaired follicular angiogenesis and oxygenation and increasing pregnancy implantation by improving endometrial receptivity by adequate vascularisation and oxygenation in unexplained infertility patients respectively [15,16]. Although new; these trials offer hopeful results for future infertility patients.

Principal mechanisms of HBO are based on intracellular generation of reactive species of oxygen and nitrogen by causing oxidative stress. Reactive oxygen species (ROS) are recognized to play a central role in cell signal transduction cascades or pathways, for a variety of growth factors, cytokines, and hormones. Scavenging antioxidants combat the overproduction of reactive species like vitamine E and C, glutation [10,17]. Recently, some new data about the role of oxidative stress and antioxidants in modulation of ovarian techal-interstitial cells have been enlightened. First, it was demonstrated that antioxidants inhibited proliferation of theca-interstitial cells then new findings added that antioxidants with distinctly different mechanisms of action induce a series of events consistent with the process of apoptosis in ovarian mesenchyme [18,19]. As PCOS is a condition associated with excessive growth and activity of theca-interstitial cells, these findings bring the question of the role of oxidative stress and antioxidants in PCO disease.

Treatment of human PCOS is symptomatic, lifestyle measures such as diet and exercise (or both) could play an important role in optimising healthy weight, improving underlying hormonal disturbances, prevention of future reproductive and metabolic complications and improving quality of life. Lifestyle interventions are recommended first-line in an international position statement on PCOS and present a cost effective initial treatment strategy compared to surgical and pharmacological options [20]. In the recent Cochrane review the positive effects of lifestyle treatment on anthropometric (abdominal adiposity, adiposity distribution), reproductive (biochemical and clinical hyperandrogenism) and metabolic features (markers of insulin resistance) were confirmed with medium or long term lifestyle measures [21].

In the present study, we tried to find a new, alternative treatment modality for PCO disease, considering the different effects of hyperoxia on different tissues, mentioned above. For this, we tested the hypothesis that 6 weeks of HBO administration on polycystic rat ovary would make an improvement or reversal of PCO state. This was carried out by studying the effect of HBO on ovarian morphology including different folicular stages.

## Methods

### Animals

80 young, cyclic female Wistar-Albino rats (250-300 g) were used for the experiment. All rats were provided by Experimental Medicine Research Center (DETAM) of Istanbul University and housed in the Animal Laboratory of the same centre. They were caged in a controlled environment of 22°C with 12 h light/dark cycles and a humidity range between 40 and 60%. Standard rat feed and reverse-osmosis-purified water were provided ad libitum. All rats were allowed to have 1 week of acclimation to this environment before the experiment. This study approved by 'Animal Studies Comittee' at DETAM Unite of Istanbul University, Istanbul and all investigations complied with the 1996 National Academy of Science's Guide for Care and Use of Laboratory. Eighty animals were divided into four experimental groups: group 1, control (n = 20); group 2, PCO only group (n = 20); group 3, PCO+HBO group (n = 20) and group 4, HBO only group (n = 20).

### PCO induction

Young cyclic Wistar females were taken and had free access to pelleted rat food and water. All animals were displaying at least 2 consecutive normal 4-day estrous cycles were used in this study. Estrous cycles prior to and after treatment were monitored by daily examination of vaginal smears.

To induce a well defined PCO, 4 mg im injection of EV (Sigma, Riedeldehaen, Germany) was administered in 0.2 ml of sesame oil. Forthy rats those in the PCO groups received EV and the remaining rats (also in estrus) those in the oil groups were injected 0.2 ml oil. A duration of 6 weeks was chosen because a single injection of EV induces, after a lag period of 4-6 week, a chronic anovulatory PCO condition in adult rats [6,7].

### HBO

After full PCO state was obtained (after six weeks from the EV or oil injection), the rats were allowed to take six weeks of HBOT. We have chosen 6 weeks of HBOT depending on protocols of Department of Underwater and Hyberbaric Oxygen Unite for chronic diseases protocol for rats.

Hyperbaric Chambers and standard protocols used for rats that is 2.5 atm pressure, totally 60 minutes once a day protocol is used [8,9]. The chamber was ventilated with 100% oxygen at a flow rate of 20 L/min to minimize CO_2 _accumulation. Rats were then slowly decompressed at a rate of 1 ATA/min at Istanbul University Underseas and Hyperbaric O2 Department.

The rats were decapitated after HBOT finished, independent of cycle day. Before decapitation, all rats were anaesthetised with an i.p. administration 50 mg/kg ketamine hydrochloric acid (Ketalar; Eczacibasi Warner-Lambert Ilac Sanayi, Levent, Istanbul, Turkey) and 7 mg/kg Xylazine hydrochloric acid (Rompun, Bayer Sisli, Istanbul, Turkey)

### Ovarian morphology and quantitative analysis of follicle populations

#### Tissues

After death of one rat, rats were analysed, independent of cycle day when full characteristic changes of ovaries ensued, the ovaries were removed. Both ovaries were weighed and fixed in buffered 4% formaldehyde for at least 24 h. Thereafter, the samples were dehydrated and embedded in paraffin to await morphological analyses by the same author.

Only the number of follicles containing an oocyte with a nucleus were classified as healthy and counted. If ovum degeneration or at least one pyknotic granulosa cell was observed, the follicle populations were classified as being atretic. Primary and secondary follicles in each section were enumerated only if the nucleolus of the ovum was visible. The enumerated follicle was then classified as healthy or atretic. Primary follicles were described as those having an intact, enlarged oocyte with a visible nucleus and a single layer of cuboidal granulosa cells. Secondary follicles were described as follicles with two or more layers of cuboidal granulosa cells, but formation of a cavity is not evident. Tertiary or graafian follicles which was described as the presence of an antral cavity within the follicle.

Quantification analyses were performed using 8-μm-thick slices under light microscopy, adopting one section and discarding ten sections in sequence and finally resulting in 12 repetitions/ovary [22,23]. The primary, secondary, tertiary antral and atretic follicles, fresh corpora lutea were each counted that stained with haematoxylin and eosin on each ovary and numbers were given per rat. All data were analyzed under a Zeiss Axiophot II microscope (Carl Zeiss, Germany) using 20× magnification for primordial and primary follicles and 10× for others.

### Statistical analysis

All statistical evaluations were performed using the software SPSS 15.0 (SPSS Inc., Chicago, USA). The effect of the follicle counts of the ovaries after EV injection and/or HBO on were evaluated using Kruskal Wallis test. *p *< 0.05 was considered statistically significant. After Kruskal Wallis, Mann Whitney-*U *test with Benforini adjustment was applied.

## Results

**Figure 1:**
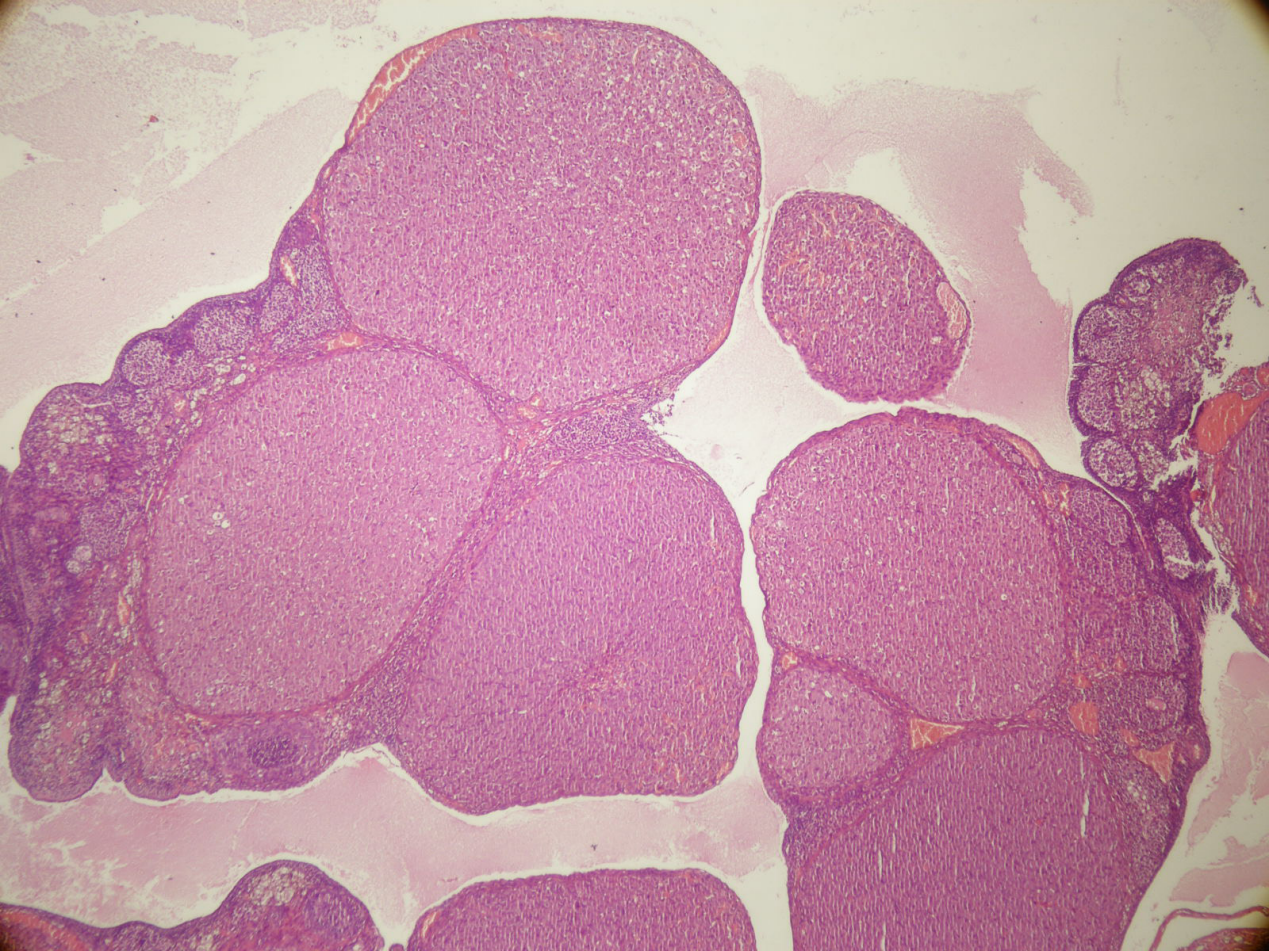
**Ovarian morphology in Control group**.

In control group (group 1), the ovaries exhibited a typically normal appearance with follicles and corpora lutea in different stages of development and regression (Figure 1). The number of atresic follicles was significantly lower and the numbers of fresh corpora luteas, tertiary follicles, secondary follicles and primary follicles were significantly higher than other groups (Table 1 and Table 2).

**Table 1:** Comparison of mean numbers of follicles

	Group 1	Group 2	Group 3	Group 4	*p*
***AF***	16.5 ± 3.7	25.4 ± 5,3	34.2 ± 8.2	29.1 ± 6.3	**0.023**

***CL***	13.5 ± 5.2	4.4 ± 3.6	3.1 ± 1,5	4.6 ± 4.2	**0.002**

***TF***	6.1 ± 2.6	2.4 ± 1.1	1.9 ± 0,8	2.1 ± 0.6	**0.014**

***SF***	13.5 ± 8.5	3.1 ± 0.9	3.2 ± 1.9	4.3 ± 0.5	**0.004**

***PF***	5.8 ± 2.3	1.6 ± 1.1	1.5 ± 1.3	1.6 ± 0.5	**0.003**

*p values of Kruskal-Wallis test

AF: atretic follicle, CL:fresh corpus luteum, TF: tertiary follicle, SF:secondary follicle, PF: primary follicle.

**Table 2:** Comparison of p values of groups regarding follicle types

*P*	AF	CL	TF	SF	PF
*Group 1/Group 2*	0.028	0.021	0.012	0.008	0.013

*Group 1/Group 3*	0.011	0.004	0.002	0.012	0.021

*Group 1/Group 4*	0.024	0.034	0.003	0.019	0.003

*Group 2/Group 3*	0.037	NS	NS	NS	NS

*Group 2/Group 4*	NS	NS	NS	NS	NS

*Group 3/Group 4*	NS	NS	NS	NS	NS

*p values of Mann Whitney-*U *test with Benforini adjustment for multiple comparisons.

NS: nonsignificant

AF:atretic follicle, CL:fresh corpus luteum, TF:tertiary follicle, SF:secondary follicle, PF:primary follicle

**Figure 2:**
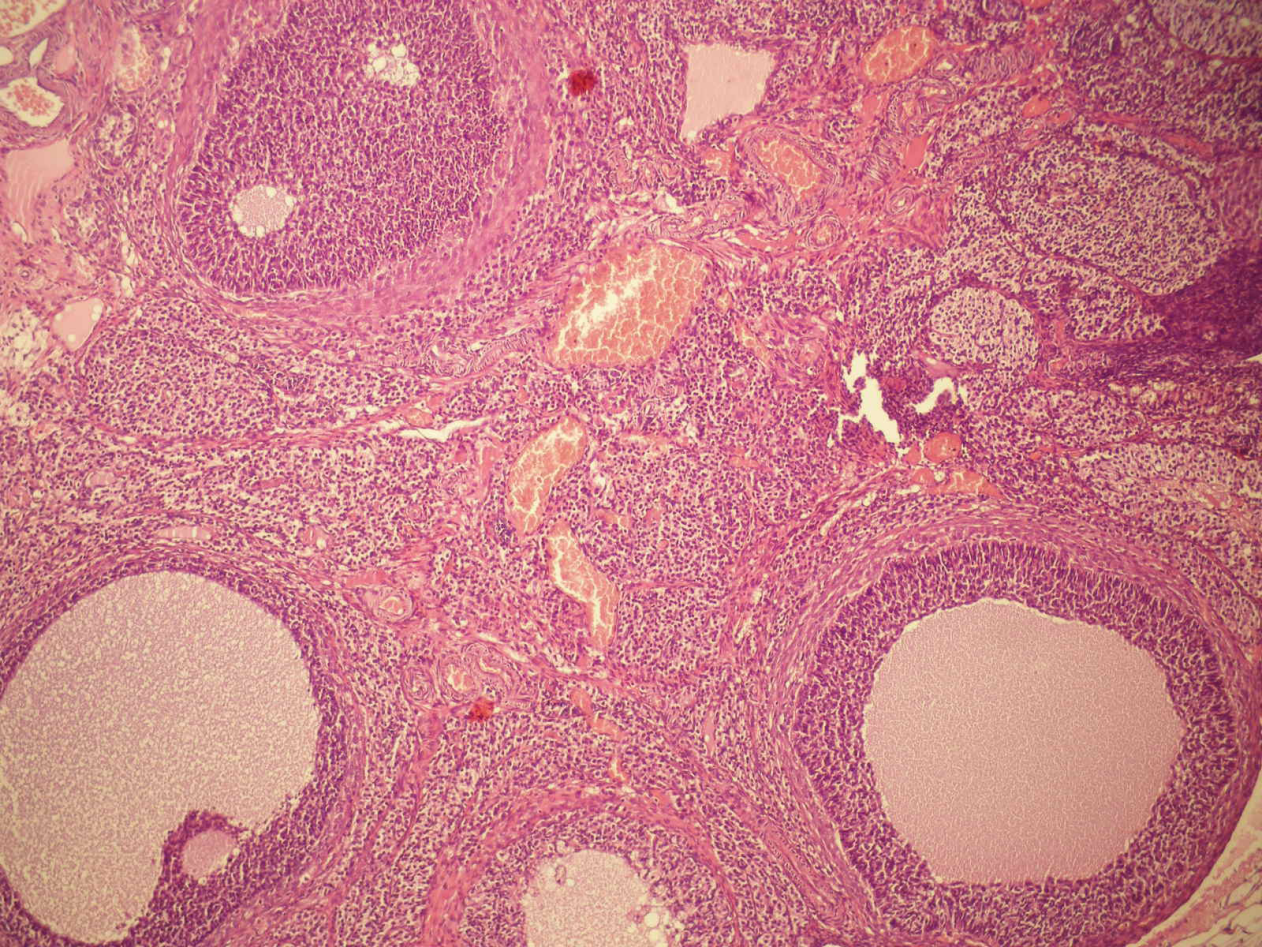
**Cystic Follicles of Polycystic ovary (PCO) group**.

In PCO only group (group 2), the ovaries displayed typical PCO-like changes (Figure 2); mean numbers of tertiary, secondary and primary follicles were significantly lower compared with the control group (Table 1 and Table 2). Typically, the number of atretic follicles were significantly higher in the PCO only group compared to control and very few fresh corpora lutea was observed.

**Figure 3:**
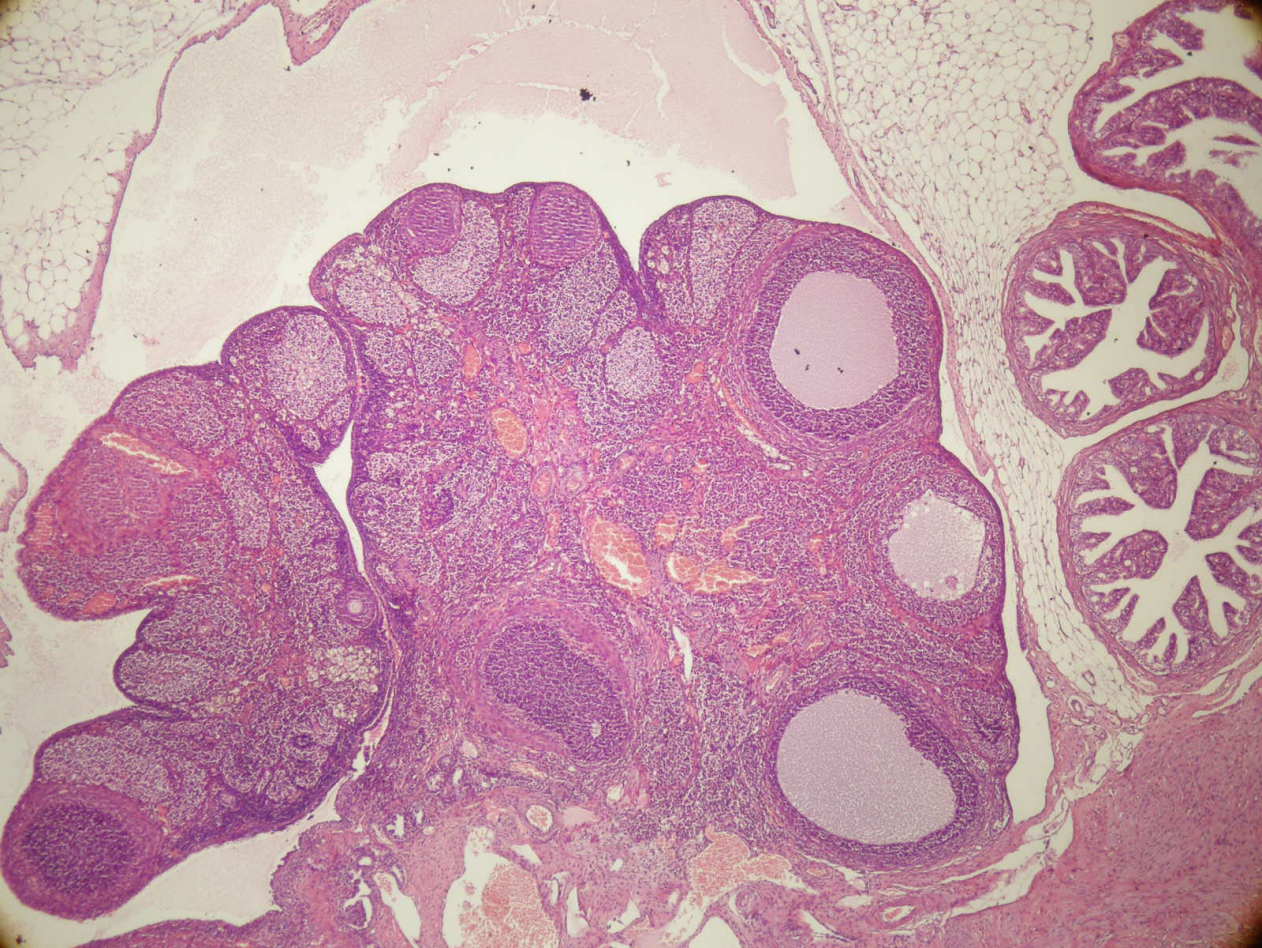
**Atretic and cystic follicles in PCO+HBO group**.

In the PCO+HBO group (group 3), there were significantly higher atretic follicles (Figure 3) compared to the PCO only group (*p *= 0.037). Although number of atretic follicles in group 3 seems to be more than group 4, the difference was not statistically significant (Table 2).

**Figure 4:**
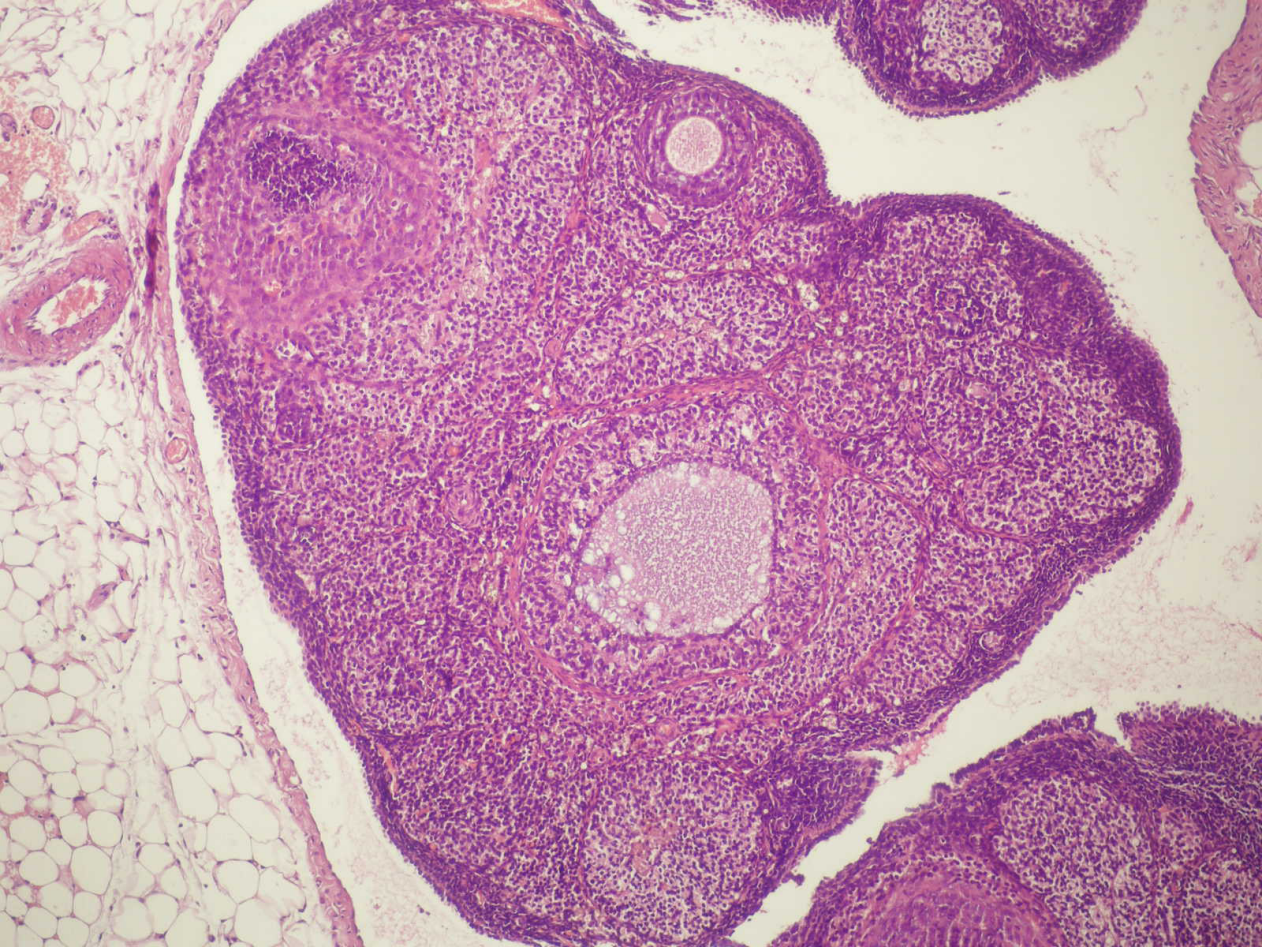
**Marked atretic follicles in HBO group**.

In the only HBO group (Group 4), there were significantly higher atretic follicles compared to group 1 (*p *= 0,024). There were significantly lower fresh corporea lutea (*p *= 0,034) and healthy growing follicles (Table 2). Although the number of atretic follicles were seems to be higher (Figure 4), it did not differ significantly compared with group 3.

## Discussion

Cystic ovarian disease and/or PCOS are disorders of the reproduction that affect bovine, ovine, caprine and porcine species and even human beings [1,2]. Several experimental models for PCO have been developed in rats; of these EV cause a sudden appearance of PCO due to disturbances in metabolic and physiologic processes [6,7].

Many studies were established such as electroacupuncture, hemohim, Korean red ginseng extract, herbal medicine, exercise assessing the effect of different therapies on EV induced PCOS rats [24-27]. Some of them found improvement in ovarian morphology of PCO state, and some of them did not. The difference of our study from those is that; they investigated the inhibition of PCO state starting therapies on the same day with EV injection, but we wanted to investigate the improvement in ovarian morphology of PCO state by starting HBOT after PCO morphology was fully established, rather than to investigate the inhibition of formation of PCO morphology.

The application of HBO ensures a normal supply of molecular oxygen to mitochondria, transported from the lungs to the endometrial cells or to ovarian tissue in a physically dissolved form in body fluids. Ultimately the increases in dissolved oxygen generated by HBO have several physiologic effects that can alter tissue responses to disease and injury [8,15]. In our study, after PCO formation, six weeks of HBO treatment, 60 minutes once a day protocol did not reverse but in contrast, aggravated the atresia of follicular growth in the rat ovaries, significantly. HBOT of healthy rat ovary increased the young follicular atresia and decreased the healthy growing follicles and also decreased the fresh corporea lutea significantly. The mean number healthy growing follicles were found lower than control and PCO groups but similar with PCO+HBO group. So this forced us to think that HBO administration created the same effect on ovarian histology like EV. Significantly, the mean number of atresia was higher in HBO+PCO group compared to PCO group, we think that the accelerated atresia is due to HBOT and HBO did not help to improve PCO state in the ovaries but inspite aggravated it. When we reviewed our results with rat studies about HBOT, we came to know that HBO administration also led to unwelcome side-effects, such as oxidative stress and/or oxygen toxicity, the formation of ROS can cause cellular damage through the oxidation of lipids, proteins and DNA [28]. One study assessing oxidative stress levels in rats following exposure to oxygen at 3 atm for 0-120 minutes exhibited a clear relationship of HBO-induced oxidative action to exposure time. This action was most pronounced from 90 to 120 minutes of exposure [29]. Another study on the effects of HBO in rats revealed that after 2 hours of HBO exposure at 3 ATA, levels of the oxidative stress markers elevated in lung, brain and erythrocytes [30]. Repetitive treatment with HBO was proved to cause oxidative stress in critical tissues including the rat brain, lymphocytes. Malondialdehyde levels and other oxidative stres markers were found to be significantly increased in the therapies after 20 day to 40 day session groups [31]. Although HBO-mediated free radicals were accepted to be responsible for the benefits of this therapeutic modality, especially in cases with prolonged exposure, possible injurious effects of supranormal values of bio-oxidative products need to be considered. Our application of 6 weeks HBO might be too much regarding exposure time and duration of it for PCO rats which is a multi-system disease that already carries underlying metabolic pathologies. Confirming this, Takahashi et al. found a biphasic effect of the generation of superoxide radicals on proliferation consistent with the concept that while moderate oxidative stress may induce cell growth, very high levels of ROS were cytotoxic and led to cell, tissue damage and apoptotic cell death [32]. Although we did not measure oxidative stress markers, all these aforementioned data may help to explain why we found aggrevation of atresia rather than reversal of it. As we did not measure the VEGF levels we can't declare clearly if modulated mechanisms of angiogenesis were causative.

Although human PCO is not the same as rat model, the oxidative effects of HBO have been investigated both in animals and human [29,30]. The protocols are consistent with the principles of partial pressures reaching that tissue. Henry's law states that the amount of gas dissolved in a liquid or tissue is proportional to the partial pressure of that gas in contact with the liquid or tissue. This is the basis for increased tissue oxygen tensions with HBOT [8,9].

The clinically approved maximum pressure and duration of HBO exposure are 3 ATA and 120 minutes, respectively although the most commonly-used protocol for standard therapeutic purposes is slightly lower (1.8-2.8 ATA for 60-90 min) [9,28]. We applied chronic diseases standard HBO protocol (2.5 atm, 60 minute once a day) for our rat PCO model, considering the PCO as multi-systemic and chronic disease. Treatment recommendations mentioned in this paper are taken from the UHMS report and are evidence-based [8]. Protocols vary greatly, but the UHMS recommend treatments beginning at 2.0-2.5 ATA for up to 120 minutes at least once a day until no improvement in diseases are reached. When we reviewed rat HBO studies, we found there were different protocols like 2 atm, 2.8 atm/90 min for 5-40 days or 3 atm for up to 120 minutes, although guidelines vary depending on the injury [28-31]. It would be more favourable for our study if we could apply different pressures and different exposure time and analyze their effects. This will be our goal for future studies.

There is a substantial body of literature that has examined the adverse and beneficial effects of HBOT in animal models. As technology becomes more readily available to clinical practice and more clinical trials are performed to define its effectiveness, HBOT may be considered as an additional therapeutic option in many conditions. Recent studies assessed the role of HBOT in unsuccessful IVF protocols or endometrial implantation failures and found convincing results although the standard protocols are not yet established. We tried to find an alternative or adjunctive treatment modality for PCO. Depending on the tissue concentration it can either exert beneficial physiologic effects or damage cell structures. We suggest that our present HBO protocol is not applicable for treatment of PCO disease and that should be considered in infertility patients with PCO; but may be rearranged accordingly in rat PCO model which needs further studies.

Quantifying the follicles may be time consuming and not an easy way of assessing ovarian histology, this is an experimental study and we wanted to assess the details of ovarian morphology other than ovulation [22,23].

Our study is the first that HBO is used in EV induced PCO rats and control rats. Our study is privileged from others that, the long- term effect of HBO on PCOS was demonstrated. To our knowledge, there is no study in the literature evaluating the effect of HBOT on PCO.

## Conclusions

We conclude that in our experimental rat model, HBOT did not improve or reverse the PCO state in EV induced PCO rat ovaries, in contrast, after HBOT, follicular atresia increased significantly. With this protocol it seems unfavorable to administrate HBOT in PCO state. Further studies are needed to identify the accompanying factors of this result like exposure time or duration of therapy

## Abbreviations

PCOS: Polycystic Ovary Syndrome; HBO: Hyperbaric Oxygen; HBOT: Hyberbaric Oxygen Therapy; UHMS: Undersea and Hyperbaric Medical Society; EV: Estradiol Valerate; ROS: Reactive Oxygen Species; NIH: National Institute of Health; AF: Atretic Follicle; CL: Fresh Corpus Luteum; TF: Tertiary Follicle; SF: Secondary Follicle; PF: Primary Follicle; VEGF: Vascular Endothelial Growth Factor; IVF: İn-Vitro Fertilization; ATA: Atmosphere Absolute.

## Competing interests

The authors declare that they have no competing interests.

## Authors' contributions

AA has designed the study, YA has dealt with statistics, FC has dealt with rats, DS has assessed the microscopy, AArslan and AST has given the HBOT, MD has reviewed the drafts, NG read & approved the final version of the manuscript. All authors read and approved the final manuscript.
